# RNA Overwriting of Cellular mRNA by Cas13b-Directed RNA-Dependent RNA Polymerase of Influenza A Virus

**DOI:** 10.3390/ijms241210000

**Published:** 2023-06-11

**Authors:** Shinzi Ogasawara, Sae Ebashi

**Affiliations:** Department of Biology, Faculty of Science, Shinshu University, 3-1-1 Asahi, Matsumoto 390-8621, Nagano, Japan

**Keywords:** RNA editing, RNA-dependent RNA polymerase (RdRp), influenza A virus, Cas13, mis-splicing

## Abstract

Dysregulation of mRNA processing results in diseases such as cancer. Although RNA editing technologies attract attention as gene therapy for repairing aberrant mRNA, substantial sequence defects arising from mis-splicing cannot be corrected by existing techniques using adenosine deaminase acting on RNA (ADAR) due to the limitation of adenosine-to-inosine point conversion. Here, we report an RNA editing technology called “RNA overwriting” that overwrites the sequence downstream of a designated site on the target RNA by utilizing the RNA-dependent RNA polymerase (RdRp) of the influenza A virus. To enable RNA overwriting within living cells, we developed a modified RdRp by introducing H357A and E361A mutations in the polymerase basic 2 of RdRp and fusing the C-terminus with catalytically inactive Cas13b (dCas13b). The modified RdRp knocked down 46% of the target mRNA and further overwrote 21% of the mRNA. RNA overwriting is a versatile editing technique that can perform various modifications, including addition, deletion, and mutation introduction, and thus allow for repair of the aberrant mRNA produced by dysregulation of mRNA processing, such as mis-splicing.

## 1. Introduction

**Figure 1:**
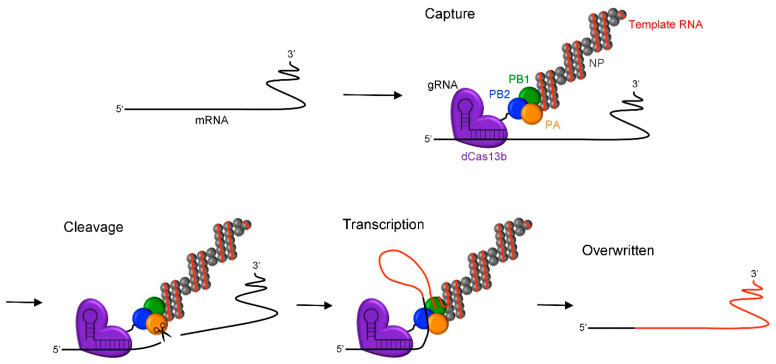
Schematic illustration of the RNA overwriting method using RdRp of IAV. RNP is composed of NP, template RNA, and RdRp consisting of PA, PB1, and PB2. RNP is induced on the target mRNA by dCas13b fused to the subunit of RdRp, followed by cleavage at the AG site by the PA subunit. The cleaved target mRNA is then used as a primer, and the PB1 subunit transcribes the template RNA. Abbreviations: RdRp, RNA-dependent RNA polymerase; IAV, influenza A virus; RNP, ribonucleoprotein; NP, nucleoprotein; PA, polymerase acidic; PB1, polymerase basic 1; PB2, polymerase basic 2; dCas13b, catalytically inactive Cas13.

mRNA undergoes post-transcriptional modifications, such as splicing, adenosine-to-inosine (A-to-I) conversion, and methylation, generating protein diversity and regulating RNA stability and translation. Dysregulation of mRNA processing can lead to various diseases, including cancer, which are called RNA diseases [1,2]. Recently, gene therapy using RNA editing technology to correct abnormal mRNA resulting from dysregulation of mRNA processing has attracted attention. Genome editing techniques, including zinc finger nucleases (ZFN) [3], transcription activator-like effector nucleases (TALEN) [4,5], and the widely known CRISPR/Cas9 system [6,7], can directly modify DNA sequences and have brought significant benefits to the life sciences through their application in various fields. However, the edited sites become permanent, leading to serious consequences if off-target editing occurs, resulting in undesired alterations to the genome, which is a critical issue in gene therapy. In contrast, RNA editing modifies mRNA, allowing the editing of genetic information without damaging the genome. As a result, even in the rare event of off-target editing, the issue can be resolved upon mRNA degradation [8]. Although the effects of RNA editing are transient, by regulating the expression duration of editing enzymes and the degradation rate of mRNA, the effects can be controlled over a period ranging from several hours to a few months. In contrast to methods combining the knockdown of target mRNA and transfection of alternative mRNA, RNA editing is advantageous because it directly edits the target mRNA, which is simpler and preserves the spatiotemporal dynamics of gene expression. The most advanced RNA editing technique involves the A-to-I conversion using adenosine deaminase acting on RNA (ADAR) [9]. Stafforst et al. (2012) reported a method to convert specific adenosines to inosines in target mRNA by generating a conjugate of guide RNA (gRNA) and ADAR in cells via a snap-tag [10]. Henceforth, various methods utilizing bacteriophage λ N protein (λn) [11,12], MS2 coat protein (MCP) [13,14], Cas proteins [15], and antisense oligonucleotides (ASOs) [16,17,18,19,20,21] have been reported for directing ADAR to target mRNA. Among these, Cas proteins to recruit editing enzymes in order to target mRNA have been applied to various RNA editing techniques, such as splicing alteration [22] and site-specific methylation [23,24]. RNA editing technology utilizing ADAR is the most promising approach; however, it is limited because it can only perform point editing from A to I, and thus cannot repair mRNA with significant defects caused by mis-splicing [25]. Recently, we reported a novel RNA editing technique called “RNA overwriting” using the RNA-dependent RNA polymerase (RdRp) of the influenza A virus (IAV) [26]. RdRp of IAV mediates the editing of host mRNA via the cap-snatching mechanism, thereby facilitating the production of viral proteins [27]. The RdRp complex is composed of three subunits, namely polymerases acidic (PA), polymerases basic 1 (PB1), and polymerases basic 2 (PB2). In the cap-snatching mechanism, the cap-binding domain of PB2 first binds to the 5′-cap of host mRNA. Subsequently, the nuclease domain of PA cleaves the mRNA at an AG site located 8–14 nucleotides downstream from the 5′-cap. Finally, using the cleaved mRNA as a primer, PB1 transcribes viral mRNA after the AG site. At this stage, forming the ribonucleoprotein (RNP) containing nucleoprotein (NP) is essential to stably bind the viral RNA (vRNA) to RdRp [28]. In RNA overwriting, the target RNA is snatched at any site by recruiting RNP to the desired editing location using guide RNA (gRNA). Previously, we demonstrated RNA overwriting using λn-fused recombinant RdRp targeting short synthetic RNAs in vitro. In this study, we report RNA overwriting in living cells using catalytically inactive Cas13b (dCas13b)-directed RdRp. (Figure 1).

## 2. Results and Discussion

### 2.1. Investigation of PB2 Mutant Lacking the Cap-Binding Ability

**Figure 2:**
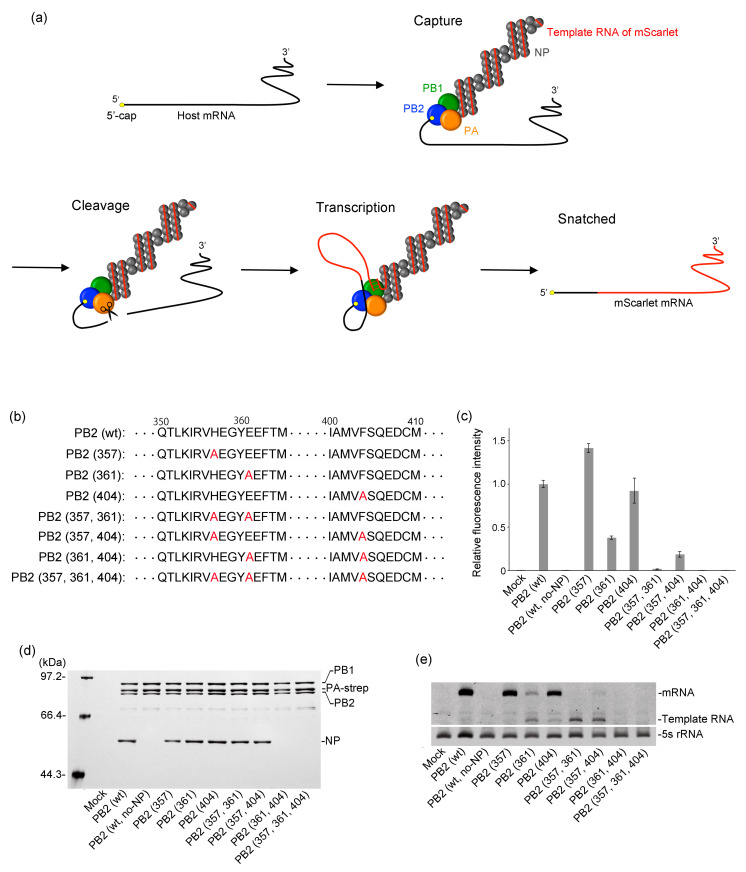
(**a**) Schematic illustration of the cap-snatching mechanism by RdRp of IAV. As the mScarlet-I template RNA is incorporated into the RNP instead of the viral RNA (vRNA), cap-snatching leads to the production of red fluorescence. (**b**) Partial amino acid sequences for the cap-binding domain of the wild-type (wt) and mutant PB2 subunits. The red characters indicate alanine substitutions. (**c**) The graph presents the mean ± standard deviation from three independent assays (n = 3). The plasmids to make up RNP were co-transfected into HEK293T cells. At 48 h post-transfection (hpt), the fluorescence intensity of cell lysates was measured using a microplate reader. (**d**) The gel image of the RNP formation assay. RNPs containing PA-strep were purified using Strep-Tactin XT and analyzed using 7.5% SDS-PAGE. (**e**) The level of mRNA and template RNA was analyzed by the primer extension assay. Ribosomal 5S rRNA was used as loading control.

The wild-type RdRp performs editing of viral mRNA by targeting host cell mRNAs through a cap-snatching mechanism (Figure 2a). Therefore, using a PB2 mutant reduced cap-binding ability is necessary to prevent cap-snatching when using RdRp for RNA overwriting. However, the introduction of mutations into PB2 must retain its transcriptional activity. Alanine scanning has identified several amino acids within the PB2 cap-binding domain (residues 318–483), contributing to binding to the 5′-cap of mRNA [29,30]. Therefore, we focused on three alanine substitutions, H357A, E361A, and F404A, and examined the cap-binding ability of all combinations of PB2 (357), PB2 (361), PB2 (404), PB2 (357, 361), PB2 (357, 404), PB2 (361, 404), and PB2 (357, 361, 404) (Figure 2b). In the RNP activity assay, the template RNA of the red fluorescent protein (mScarlet-I) was incorporated into the RNP instead of vRNA so that the cap-snatching efficiency could be estimated from the fluorescence intensity of mScarlet-I (Figure 2c). No fluorescence was observed in the absence of NP, indicating that RNP formation is essential for the cap-snatching. The fluorescence intensities of PB2 (357) and PB2 (404) were equivalent to or higher than that of the wild type, demonstrating that these PB2 mutants strongly bind to the 5′-cap. PB2 (361) and PB2 (357, 404) exhibited fluorescence intensities of 38% and 19%, respectively, compared to the wild type. No fluorescence was observed in PB2 (357, 361), PB2 (361, 404), and PB2 (357, 361, 404). The reduced cap-snatching efficiency in these five patterns was due to either reduced binding ability to the 5′-cap or incomplete RNP formation; thus, we performed the RNP formation assay. RNPs were purified from HEK293T cell lysates using a strep-tag fused to the C-terminus of PA and analyzed using SDS-PAGE (Figure 2d). Noticeable bands corresponding to PA, PB1, and PB2 were observed in all patterns, indicating the formation of stable RdRp complexes. NP bands were observed in PB2 (357), PB2 (361), PB2 (404), PB2 (357, 361), and PB2 (357, 404). In contrast, no NP bands were observed in PB2 (361, 404) and PB2 (357, 361, 404), indicating that RdRp containing PB2 (361, 404) and PB2 (357, 361, 404) does not form RNP. Moreover, we confirmed via an electrophoretic mobility shift assay (EMSA) that PB2 mutants weaken the interaction between RdRp and 5′-cap (Figure S5). These results showed that the reduced cap-snatching efficiency in PB2 (361), PB2 (357, 361), and PB2 (357, 404) occurred due to the suppressed binding ability of the PB2 to the 5′-cap. In contrast, the reduced cap-snatching efficiency in PB2 (361, 404) and PB2 (357, 361, 404) happened due to both weak cap-binding affinity and incomplete RNP formation. Therefore, PB2 (361, 404) and PB2 (357, 361, 404) are unsuitable for RNA overwriting because transcription does not occur when RNP formation is incomplete. Finally, we measured the mScarlet-I mRNA and template RNA using the primer extension assay. The mRNA levels showed a trend similar to the fluorescence intensity observed in the RNP activity assay (Figure 2e). Because RNP replicates the template RNA, if RNP is formed, the template RNA will be amplified and appear as a clear band. In PB2 (361, 404) and PB2 (357, 361, 404), no bands corresponding to template RNA were observed, indicating that RdRp containing PB2 (361, 404) and PB2 (357, 361, 404) does not form RNP. For PB2 (357, 361), although no bands were observed for mRNA, remarkable bands were detected for template RNA, suggesting that while cap-binding affinity was weakened, a complete RNP was formed. Based on these findings, we concluded that the most suitable PB2 mutant for RNA overwriting is PB2 (357, 361).

### 2.2. Influence of Fusing dCas13b with RdRp Subunits on RNP Activity

**Figure 3:**
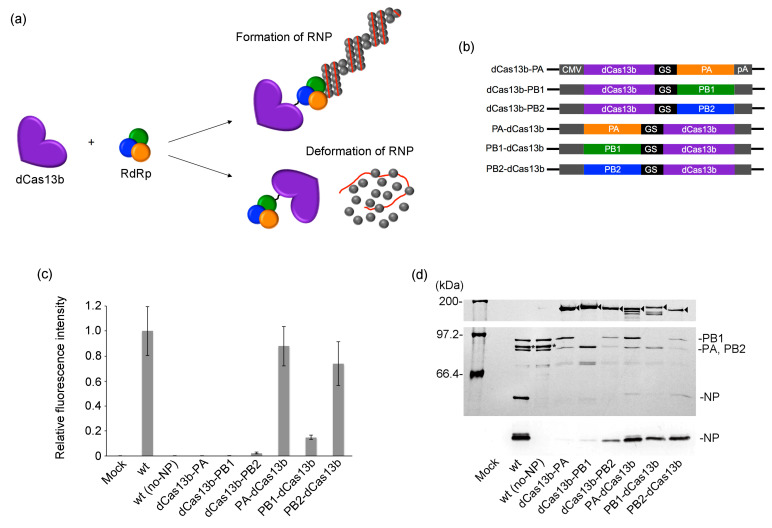
(**a**) Illustration depicting the fusion of the RdRp subunit with dCas13b. The accumulation of NP in the RNP varies depending on the position where dCas13b is fused. (**b**) Construction of plasmids used in this experiment. dCas13b is fused to the PA, PB1, or PB2 segment at the N- or C-terminus through a GS linker. CMV and pA represent the CMV promoter and BGH polyadenylation signals, respectively. (**c**) The graph presents the mean ± standard deviation from three independent assays (n = 3). The plasmids to make up RNP were co-transfected into HEK293T cells. At 48 hpt, the fluorescence intensity of cell lysates was measured using a microplate reader. (**d**) RNP formation assay for RNP containing the dCas13b-fused subunit. RNPs purified using Strep-Tactin XT were analyzed using 7.5% SDS-PAGE. Arrowheads represent the band corresponding to each dCas13b-fused subunit. Asterisks represent the band corresponding to PA-strep. NP was detected by Western blotting with anti-NP MAb.

Previously, the interaction system between the Box B sequence within the gRNA and the λn [31] was used to recruit RdRp to the editing initiation site of the target mRNA. However, in this study, we replaced this system with the interaction between CRISPR RNA (crRNA) and dCas13b [32] for RNA overwriting within living cells. In the Box B-λn interaction system, gRNA is exposed and rapidly degraded by cellular RNases. In contrast, in the crRNA-dCas13b system, dCas13b binds to and encapsulates gRNA, protecting it from degradation. Consequently, an improvement in editing efficiency is expected. Actually, RNA overwriting efficiency using the crRNA-dCas13b system showed a 3.7-fold increase compared to that of the Box B-λn system (Figure S1). Nonetheless, while the molecular mass of λn is 2.6 kDa, dCas13b is a considerably larger protein of 114.9 kDa that may inhibit RNP formation when fused to a subunit of RdRp (Figure 3a). Therefore, we investigated the RNP activity and formation when dCas13b was fused to the N-terminus and C-terminus of PA, PB1, and PB2 (Figure 3b). No mScarlet-I fluorescence was observed when dCas13b was fused to the N-terminus of any subunit, indicating a loss of RNP activity (Figure 3c). The RNP formation assay suggested that the loss of RNP activity was due to a significant decrease in NP accumulation within the RNP (Figure 3d). In contrast, when dCas13b was fused to the C-terminus, the fluorescence intensities of PA, PB1, and PB2 were 90, 15, and 75% compared to that of the wild type, respectively, showing varying degrees of RNP activity reduction. The decreased accumulation of NP within RNP was correlated with the reduction in RNP activity, indicating that the decrease in RNP activity was due to the incompleteness of RNP formation. RNP is stabilized by the distinct binding modes between each RdRp subunit and NP [33,34,35]; thus, the difference in RNP activity reduction depends on which subunit dCas13b is fused to. The results demonstrate that fusing dCas13b to the C-terminus of either PA or PB2 is optimal.

### 2.3. RNA Overwriting in Cells

**Figure 4:**
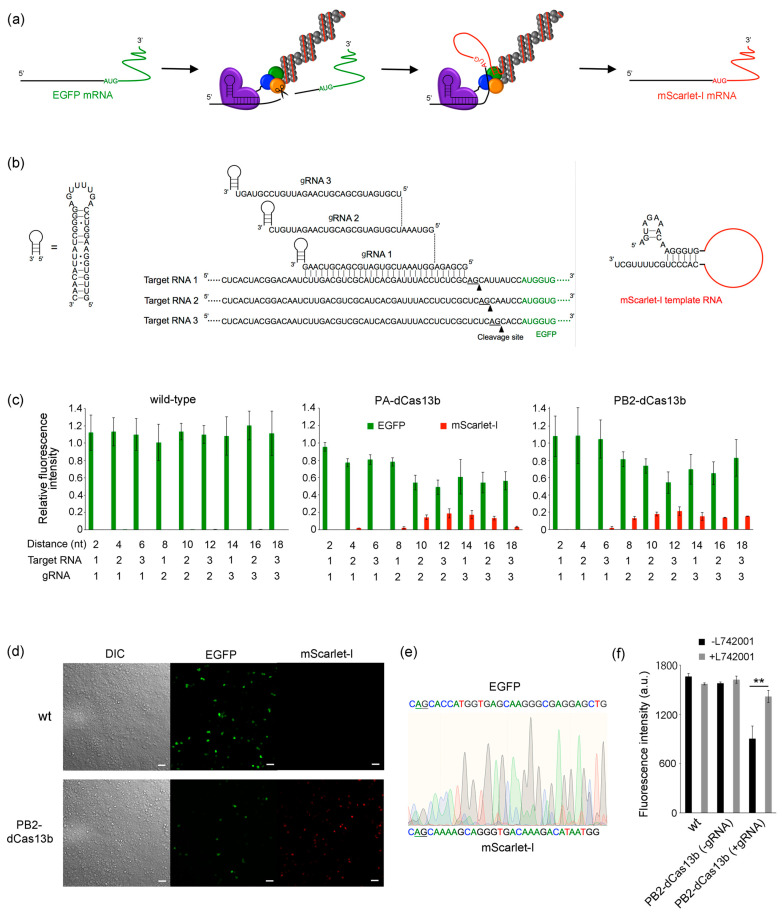
(**a**) Schematic illustration of RNA overwriting. EGFP mRNA was overwritten to mScarlet-I mRNA by RNP containing dCas13b-fused RdRp. (**b**) RNA sequences used in this experiment. By combining three types of gRNA with three target RNAs, the AG site is shifted by two nucleotides downstream from the end of duplex formation between the target RNA and gRNA, up to 18 nucleotides. (**c**) RNA overwriting using wild-type (wt), PA-dCas13b-, or PB2-dCas13b-containing RdRp. The graph presents the mean ± standard deviation from three independent assays (n = 3). The plasmids to make up RNP were co-transfected with gRNA expression plasmid and target RNA expression plasmid into HEK293T cells. At 48 hpt, the fluorescence intensity of cell lysates was measured using a microplate reader. Each fluorescence intensity was normalized to the intensity observed when RNA overwriting was performed in the absence of gRNA. (**d**) Fluorescence images for RNA overwriting by wt or PB2-dCas13b-containing RNP using gRNA 2 and target RNA 3. The images were obtained 48 hpt using a fluorescence microscope. Scale bar, 50 µm. (**e**) Sequences from RT-PCR products after overwriting by PB2-dCas13b-containing RNP using gRNA 2 and target RNA 3. The upper and lower sequences indicate the target and overwritten RNA, respectively. The underline represents the AG site, which is the initiation point of overwriting. (**f**) EGFP fluorescence in RNA overwriting with endonuclease inhibitor L742001. Asterisks denote *p*-values from Welch’s *t*-test (** *p* < 0.01).

We performed RNA overwriting in HEK293T cells using RNP containing either PA-dCas13b or PB2-dCas13b. To estimate cleavage and overwriting efficiency from fluorescence intensity, we constructed a fluorescent reporter assay system that overwrites EGFP mRNA with mScarlet-I mRNA (Figure 4a). The sequences of the gRNAs and target RNAs used in this study are shown in Figure 4b. To optimize the spacer region from the binding site of dCas13b to the editing initiation point, three types of gRNA and target RNA were prepared. By combining them, the AG sites within the target RNA shifted two nucleotides, up to 18 nucleotides downstream from the complementary sequence with the gRNA. When using wild-type RNP, no decrease in EGFP fluorescence or appearance of mScarlet-I fluorescence was observed, indicating that RNA overwriting did not occur (Figure 4c). In contrast, when RNP containing either PA-dCas13b or PB2-dCas13b was employed, decreased EGFP fluorescence and detectable mScarlet-I fluorescence were observed. The RNP containing either PA-dCas13b or PB2-dCas13b showed minimal cleavage within eight nucleotides from the end of duplex formation between the target RNA and gRNA, and cleavage efficiency increased further downstream. The RNP containing PA-dCas13b maintained approximately 50% cleavage efficiency up to 18 nucleotides downstream. In contrast, the RNP containing PB2-dCas13b exhibited 46% cleavage efficiency at 12 nucleotides downstream, with declining efficiency thereafter. Overwriting was observed from 10 to 16 nucleotides downstream for PA-dCas13b-containing RNP and 8 for PB2-dCas13b-containing RNP, with the latter showing a broader range. Moreover, using PB2-dCas13b-containing RNP with a 12-nucleotide downstream AG site as the editing start point showed the highest efficiency at 21%. Considering the 3D structure of the RdRp, the C-terminus of PA is located directly behind the entrance to the polymerase core. Therefore, when using RdRp containing PA-dCas13b, the cleaved 3′-end of the mRNA has difficulty reaching the polymerase core, resulting in decreased overwriting. Therefore, using PB2-dCas13b-containing RNP with a 12-nucleotide downstream AG site as the editing start point from the end of the gRNA complementary sequence provides the best cleavage and overwriting efficiency (Figure 4d). The overwriting efficiency peaked at 48 h post-transfection (hpt) (Figure S6). Generally, transient protein expression via plasmids peaks around 48 hpt; therefore, the decrease in overwriting efficiency after 48 hpt likely results from a reduction in the expression of the editing enzyme. The sequence of the mRNA after overwriting was confirmed by sequencing (Figure 4e and Figure S7). Two overlapping spectra were observed after the AG site, one corresponding to EGFP and the other to the overwritten mScarlet-I sequence. To confirm whether the decreased EGFP fluorescence was due to the cleavage of the target mRNA by the PA subunit, we conducted RNA overwriting with the addition of the endonuclease inhibitor L742001 against PA [36] (Figure 4f). The recovery of EGFP fluorescence upon L742001 addition indicated that the decreased EGFP fluorescence intensity was not because of dCas13b-mediated translation inhibition or mRNA degradation by intrinsic nucleases but was caused by cleavage by the endonuclease activity of the PA subunit.

### 2.4. RNA Overwriting within an Open Reading Frame

**Figure 5:**
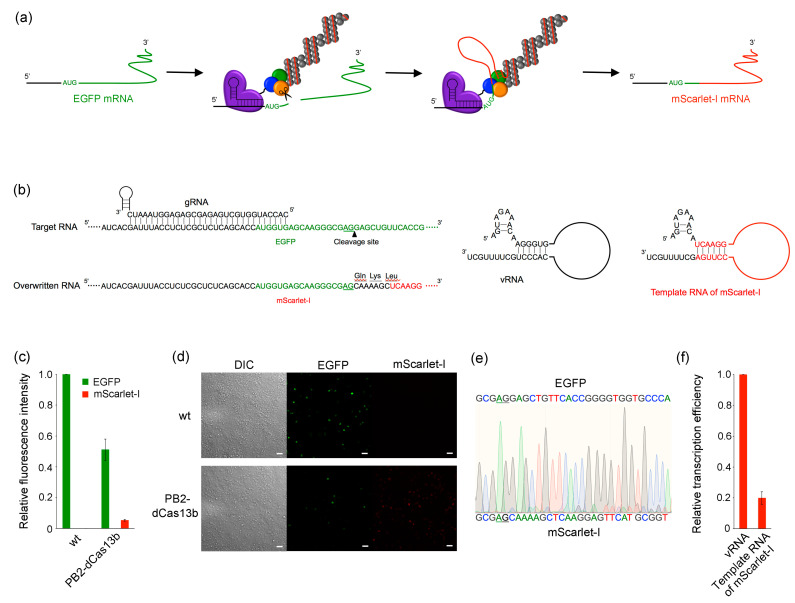
(**a**) Schematic illustration of RNA overwriting within the ORF. (**b**) RNA sequences used in this experiment. Due to the utilization of the RNA motif of the vRNA at the 3′-end of the template RNA, the sequence CAAAAGC was inserted immediately after the AG site in the overwritten RNA sequence, resulting in the introduction of a Gln-Lys-Leu mutation. (**c**) RNA overwriting using wild-type (wt) or PB2-dCas13b-containing RNP. The graph presents the mean ± standard deviation from three independent assays (n = 3). At 48 hpt, the fluorescence intensity of cell lysates was measured using a microplate reader. (**d**) Fluorescence images for RNA overwriting by wt or PB2-dCas13b-containing RNP. The images were obtained 48 hpt using a fluorescence microscope. Scale bar, 50 µm. (**e**) Sequences from RT-PCR product after overwriting. The upper and lower sequences indicate the target and overwritten RNA, respectively. The underline represents the AG site, which is the initiation point of overwriting. (**f**) RNP activity assays. Replacing the original vRNA sequence with the mScaret-I sequence in the intramolecular double-stranded formation region of the template RNA leads to decreased transcription efficiency.

We performed RNA overwriting using the AG site within the open reading frame (ORF) as the editing start point (Figure 5a). We designed the gRNA such that the AG site immediately before the divergence of EGFP and mScarlet-I sequences would be the editing start point (Figure 5b). The RNA motifs at the 5′- and 3′-ends of the vRNA are crucial for RdRp to efficiently transcribe and replicate vRNA. Introducing mutations into these RNA motifs significantly decreased the transcription and replication efficiencies. Therefore, in RNA overwriting, we employed the RNA motifs of vRNA at the 5′- and 3′-ends of the template RNA. As a result, the seven nucleotides at the 3′-end of the template RNA differed from the mScarlet-I sequence, leading to mutations from GCA(Ala) to CAA(Gln), GUG(Val) to AAG(Lys), and AUC(Ile) to CUC(Leu) in the overwritten sequence. However, these mutations did not affect the fluorescence spectrum of mScarlet-I. By RNA overwriting, EGFP fluorescence decreased by 49%, and mScarlet-I fluorescence emerged (Figure 5c,d). Sequence analysis confirmed that the mRNA of EGFP was rewritten to that of mScarlet-I (Figure 5e and Figure S8). The overwriting efficiency was 5%, considerably lower in comparison to the 21% observed in the previous experiment due to the change in an intramolecular double-stranded formation region of the template RNA from the original vRNA sequence to that of mScarlet-I. The RNP activity assay revealed that changing the vRNA intramolecular double-stranded formation region to the mScarlet-I sequence reduced the transcription activity by 1/5 (Figure 5f). To achieve efficient RNA overwriting within the ORF, the design of the 3′-end RNA sequence and intramolecular double-stranded formation region of the template RNA is important. To perform RNA overwriting without introducing mutations into the overwritten mRNA sequence or limiting mutations to those that do not affect protein function, changing the 3′-end sequence of the template RNA from the original vRNA sequence is necessary. Several studies showed the impact of introducing mutations into the RNA motifs and intramolecular double-stranded formation regions at the 5′- and 3′-ends of vRNA on the transcription and replication efficiencies [37,38,39,40,41]. Some mutations can improve those efficiencies. In the future, comprehensively investigating the effects of introducing mutations into the 5′- and 3′-end sequences of vRNA on transcription and replication efficiencies will be necessary.

## 3. Materials and Methods

### 3.1. Cells

A 293T human embryonic kidney (HEK 293T) was cultured with Dulbecco modified Eagle medium (DMEM, Fujifilm, Osake, Japan) supplemented with 10% fetal calf serum (FCS, Japan Bio Serum, Fukuyame, Japan), 100 units/mL penicillin, and 100 mg/mL streptomycin (Fujifilm) at 37 °C in 5% CO_2_.

### 3.2. Construction of Plasmids

All plasmids used in this study were constructed by standard PCR and seamless cloning methods. PA, PB1, PB2, or NP segments of influenza A/Puerto Rico/8/1934 (PR8) were subcloned into pcDNA3 plasmid and designed pcDNA3-PA, -PB1, -PB2, or -NP. The plasmid of PA, PB1, or PB2 fused with the dCas13b at N- or C-terminus was obtained by cloning the sequence of dCas13b into pcDNA3-PA, PB1, and PB2. The plasmids for the PB2 mutant were prepared using a PCR-based site-directed mutagenesis method. The pPolI plasmid was constructed by inserting the synthetic human RNA polymerase I promoter (p_I_) and the mouse RNA polymerase I terminator (t_I_) sequence into pcDNA3, truncating the CMV promoter and BGH polyadenylation signal sequence. pPolI-mScarlet was constructed by inserting the mScarlet-I sequence between p_I_ and t_I_ of the pPolI plasmid in the negative-sense orientation (Figure S2). To obtain gRNA expression plasmids (phU6-gRNA), a synthetic primer containing the gRNA sequence was used in a PCR, and the resulting product was subjected to seamless cloning (Figure S3). The target mRNA expression plasmids (pcDNA3-target) were constructed by cloning the ORF of EGFP and the mouse ornithine decarboxylase (MODC) PEST sequence [42], which shortens the mRNA half-life, into pcDNA3 (Figure S4).

### 3.3. RNP Activity Assay

The HEK 293T cells were seeded one day prior to transfection on a 3.5 cm glass bottom or normal culture dish with 5 × 10^5^ cells/2 mL. A total of 0.7 μg each of the pcDNA-PA, -PB1, and -PB2 (or PB2 mutant) plasmids was co-transfected with 0.6 μg of pcDNA-NP and 0.3 μg of pPolI-mScarlet plasmids using Lipofectamine LTX (Thermo Fisher Science, Waltham, MA, USA) and Opti-MEM (Thermo Fisher Science) according to the manufacturer’s instructions. At 48 h post-transfection (hpt), red fluorescence images were taken using a fluorescence microscope (Eclipse Ti2, Nikon, Tokyo, Japan), and cells were lysed in 100 μL of lysis buffer (50 mM Tris-HCl, pH 8.0, 200 mM NaCl, 0.5% Nonidet P-40 [Sigma-Aldrich, St. Louis, MI, USA], 1 mM DTT, 1 mM PMSF, 25% glycerol, and 1× protease inhibitor cocktail [Fujifilm]) at 4 °C for 60 min. The lysates were analyzed using a fluorescence microplate reader (SH-9000, Hitachi, Tokyo, Japan).

### 3.4. RNP Formation Assay

The HEK 293T cells were seeded one day prior to transfection on a 10 cm culture dish with 4 × 10^6^ cells/10 mL. A total of 2.7 μg each of the pcDNA-PA (-dCas13b-PA or -PA-dCas13b or PA-strep), -PB1 (-dCas13b-PB1 or -PB1-dCas13b), and -PB2 (-dCas13b-PB2 or -PB2-dCas13b or PB2-mutant) plasmids was co-transfected with 2.5 μg of pcDNA-NP and 1.3 μg of pPolI-mScarlet plasmids in HEK 293T cells using Lipofectamine LTX and Opti-MEM. The sequence of twin-strep-tag (WSHPQFEKGGGSGGGSGGSAWSHPQFEK) was inserted into dCas13b-PA (or PA-dCas13b), dCas13b-PB1 (or PB1-dCas13b), and dCas13b-PB2 (or PB2-dCas13b) before (or after) the dCas13b. At 48 hpt, cells were lysed in 500 μL of lysis buffer at 4 °C for 60 min. To the lysate in 2.5 mL of binding buffer (100 mM Tris-HCl, pH 8.0, and 150 mM NaCl), Strep-Tactin XT 4Flow (IBA) (50 μL) was added and incubated at 4 °C for 3 h. The Strep-Tactin XT 4Flow was then washed three times with 3 mL of wash buffer (100 mM Tris-HCl, pH 8.0, 150 mM NaCl, 0.1% Nonidet P-40, 1 mM PMSF, and 10% glycerol). The RNP complex was released from the Strep-Tactin XT 4Flow by elution buffer (100 mM Tris-HCl, pH 8.0, 150 mM NaCl, 50 mM biotin, 0.1% Nonidet P-40, 0.1% glycerol, 1 mM DTT, 1 mM PMSF, and 1× protease inhibitor cocktail) at 4 °C for 1 h and analyzed by 7.5% SDS-PAGE, followed by silver staining.

### 3.5. Western Blot

Western blot analysis for NP was performed by electrophoretic transfer of the 7.5% polyacrylamide gel to a PVDF membrane (ATTO) in blotting buffer (25 mM Tris-HCl, pH 8.3, 192 mM glysine, 20% methanol) for 1 h. The membrane was then incubated for 1 h with blocking buffer (1× TBS, 0.1% Tween-20, 5% skim milk). After washing with TBS, the membrane was incubated with anti-NP mouse monoclonal antibody (Sino Biological, Kawasaki, Japan) diluted 1:1000 in TBS containing 0.1% Tween-20 and 5% BSA. Then the membrane was washed with TBS, followed by incubation at 4 °C overnight with HRP-conjugated goat anti-mouse IgG secondary antibody (Proteintech, Rosemont, IL, USA) diluted 1:5000 in TBS containing 0.1% Tween-20 and 5% skim milk. After washing with TBS, blots were developed via staining solution (WSE-7140 EzWestBlue W, ATTO, Tokyo, Japan).

### 3.6. Primer Extension Assay

The HEK 293T cells were seeded one day prior to transfection on a 10 cm culture dish with 4 × 10^6^ cells/10 mL. A total of 2.7 μg each of the pcDNA-PA, -PB1, and -PB2 (or PB2-mutant) plasmids was co-transfected with 2.5 μg of pcDNA-NP and 1.3 μg of pPolI-mScarlet plasmids in HEK 293T cells using Lipofectamine LTX and Opti-MEM. At 48 hpt, the total RNA was extracted using TRI Reagent (Molecular Research Center, Cincinnati, OH, USA). The mScarlet-I mRNA and template RNA were reverse transcribed using FAM-labeled primer (for mRNA: 5′-FAM-CTCGATCTCGAACTCGTGG-3′; for vRNA: 5′-FAM-AACAGTACGAACGCTCCGAG-3′). The products were analyzed by 15% denaturing PAGE containing 8 M urea in Tris-glysine buffer.

### 3.7. RNA Overwriting

The HEK 293T cells were seeded one day prior to transfection on a 3.5 cm glass bottom or normal culture dish with 5 × 10^5^ cells/2 mL. A total of 0.2 μg each of the pcDNA-PA (or -PA-dCas13b), -PB1, -PB2 (or -PB2-dCas13b), and pcDNA-NP plasmids and 0.1 μg of the pPolI-mScarlet plasmid were co-transfected with 30 ng of pcDNA-target plasmids in the presence or absence of 3.8 μg of phU6-gRNA plasmids in HEK 293T cells using Lipofectamine LTX and Opti-MEM. At 48 hpt, green and red fluorescence images were taken using a fluorescence microscope, and cells were lysed in 100 μL of lysis buffer at 4 °C for 60 min. The lysates were analyzed using a fluorescence microplate reader. To inhibit the endonuclease activity of the PA subunit, the inhibitor L742001 (Sigma-Aldrich) (21.5 μM) was added to the culture medium. For preparation of the sequencing sample, the total RNA was extracted using TRI Reagent. The extracted RNA was reverse transcribed to cDNA using RT primer (5′-CTTGTACAGCTCGTCCATGC-3′), followed by amplification by PCR using primers (forward primer: 5′-CACTACGGACAATCTTGACG-3′; reverse primer: 5′-CTTGTACAGCTCGTCCATGC-3′). The product was directly sequenced. The relative overwriting efficiency was calculated in comparison to the case when RNA overwriting was performed using plasmids for expression of mScarlet-I mRNA instead of target EGFP mRNA.

## 4. Conclusions

In summary, we performed two optimizations to generate a modified RdRp to conduct RNA overwriting within living cells. First, we searched for a mutant of PB2 that does not bind to the 5′-cap of mRNA and is capable of forming RNP for transcription and replication. We found an optimal mutant containing H357A and E361A. Second, we optimized the fusion site for dCas13b. Because dCas13b is large, it inhibited RNP formation when fused to the N-terminus of PA, PB1, and PB2 or the C-terminus of PB1. However, when dCas13b was fused to the C-terminus of PA or PB2, RNP was formed, with its activity maintained at 75–90% compared to the wild type. Thus, we demonstrated the in-cell overwriting of EGFP mRNA with mScarlet-I mRNA using a modified RdRp containing either PA-dCas13b or PB2-dCas13b. When the editing was initiated at the AG site 12 nucleotides downstream of the double-stranded region with gRNA using the PB2-dCas13b-containing RNP, the highest editing efficiency was observed, cleaving 46% of EGFP mRNA and overwriting 21% with mScarlet-I mRNA. Finally, RNA overwriting was performed with the editing start point within the ORF. While the cleavage efficiency of the target mRNA was 51%, the overwriting efficiency was 5% due to the change in the intramolecular double-stranded region of the template RNA from the vRNA sequence to that of mScarlet-I. Moreover, mutations were introduced into the overwritten sequence because the RNA motif of vRNA was required at the 3′-end of the template RNA. Compared to the RNA editing technology using ADAR, which can only perform A-to-I point editing, RNA overwriting is a versatile editing technique that can perform various modifications, including addition, deletion, and mutation introduction. Therefore, our technology is expected to be applicable in gene therapy to treat RNA diseases caused by dysregulation of mRNA processing, such as mis-splicing. For instance, cancer cells often contain abnormal mRNA of p53, produced by mis-splicing of the TP53 tumor suppressor gene [43]. Our technology could potentially enable the repair of such aberrant mRNA. However, applying our method to gene therapy presents two challenges that must be addressed. The first is the limitation in the design of the template RNA sequence, as mentioned above. In the future, we need to introduce all conceivable combination mutations to the terminal sequence of the template RNA and systematically investigate their impact on transcriptional activity of RNP to determine the permissible sequences. The second challenge involves the delivery of the editing enzymes, gRNA, and template RNA to target cells. The total base number of the genes comprising the modified RNP used for RNA overwriting is approximately 13.5 kbp, making commonly used viral vectors such as adenoviruses, adeno-associated viruses, and lentiviruses unsuitable. Therefore, we are considering using a simplex herpes virus vector, which can accommodate < 150 kbp of foreign genes, to deliver the modified RNP to target cells [44].

## Data Availability

Not applicable.

## Supplementary Materials

The supporting information can be downloaded at: https://www.mdpi.com/article/10.3390/ijms241210000/s1.
