# Supplementary material for: RNA Overwriting of Cellular mRNA by Cas13b-Directed RNA-Dependent RNA Polymerase of Influenza A Virus
Source: Int J Mol Sci. 2023 Jun 11;24(12):10000. doi: 10.3390/ijms241210000 (PMC10298375; doi:10.3390/ijms241210000)
Supplement: Supplementary file 1 [file ijms-24-10000-s001.zip › ijms-2417650-supplementary.pdf]

Supplementary materials

RNA overwriting of Cellular mRNA by Cas13b-directed RNA-dependent RNA Polymerase of Influenza A Virus

Shinzi Ogasawara and Sae Ebashi

Department of Biology, Faculty of Science, Shinshu University, 3-1-1 Asahi, Matsumoto, Nagano, 390-8621, JAPAN

Contents

1. Comparison of RNA overwriting efficiency between λn and dCas13b
2. Plasmids
3. EMSA for RdRp containing PB2 mutants
4. Time-course of RNA overwriting
5. Sequencing data

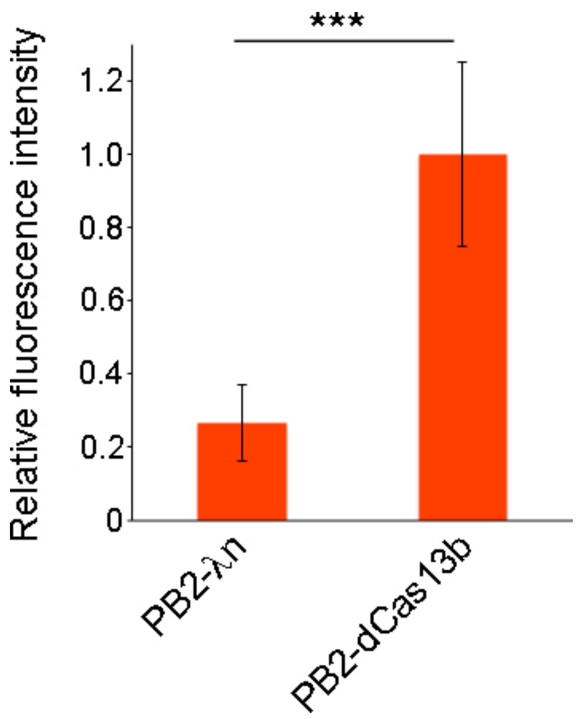

Figure S1. The mScarlet-I fluorescence in RNA overwriting by PB2-λn- or PB2-dCas13b-containing RNP using gRNA 2 and target RNA 3. The graph presents the mean ± standard deviation from three independent assays (n = 3). At 48 hpt, the fluorescence intensity of cell lysates was measured using a microplate reader. Asterisks denote *p*-values from Welch's *t*-test (*** $p < 0.001$).

5'-CGCGATGTACGGGCCAGATATACGCGTTAGATCATACATGGAAGCACGGGCCGGCC

human RNA polymerase I promoter

CCTGCGTGTGGCCAGGGCGGCCGGGAGGGCTCCCCGGCCCGGCGCTGTCCCCGCGT

GTGTCCTTGGGTTGACCAGAGGGCCCCGGGCGCTCCGTGTGTGGCTGCGATGGTGGC

GTTTTTGGGGACAGGTGTCCGTGTCGCGCGTCGCCTGGGCCGGCGGCGTGGTCGGTG

ACGCGACCTCCCGGCCCCGGGGAGGTATATCTTTCGCTCCGAGTCGGCATTTTGGGCC

GCCGGGTTATTAGTAGAAACAAGGGTGTTTTTTCATGAAGGACAAGCTATCACTTGTACA

GCTCGTCCATGCCGCCGGTGGAGTGGCGGCCCTCGGAGCGTTCGTACTGTTCCACCA

CGGTGTAGTCCTCGTTGTGGGAGGTGATGTCCAACTTGCGGTCGACGTTGTAGGCGCC

mScarlet-I template

GGGCATCTGCACGGGCTTCTTGGCCTTGTAGGTGGTCTTGAAGTCCGCCAGGTAGCGG

CCGCCGTCCTTCAGGCGCAGGGCCATCTTAATGTCGCCCTTCAGCACGCCGTCCTCGG

GGTACAACCGCTCGGTGGACGCTTCCCAGCCCATTGTCTTCTTCTGCATTACGGGGCC

GTCAGGAGGGAAGTTGGTGCCGCGGAGCTTCACCTTGTAGATCAGGGTGCCGTCCTCC

AGGGAGGTGTCCTGGGTCACGGTCACGGCGCCGCCGTCCTCGAAGTTCATCACGCGC

TCCCACTTGAAGCCCTCGGGGAAGGACTGCTTATAGTAGTCGGGGATGTCGGCGGGGT

GCTTGATGAAGGCCCTGGAGCCGTACATGAACTGAGGGGACAGGATGTCCCAGGAGAA

GGGCAGGGGGCCACCCTTGGTCACCTTCAGCTTGGCGGTCTGGGTGCCCTCGTAGGG

GCGGCCCTCGCCCTCGCCCTCGATCTCGAACTCGTGGCCGTTCATGGAGCCCTCCATG

TGCACCTTGAACCGCATGAACTCCTTGATCACTGCCTCGCCCTTGCTCACCATTATGTC

mouse RNA polymerase I terminator

TTTGTCACCCTGCTTTTGCTCCCCCCCAACTTCGGAGGTCGACCAGTACTCCGTCAATG

GACTCTGGAATCTTCTGAGGCGGAAA-3'

Figure S2 Partial DNA sequence of pPolI-mScarlet to express mScarlet-I template RNA.

human U6 promoter

5'-GCATACTCCAGCGGATCGATCCAAGGTCGGGCAGGAAGAGGGCCTATTTCCCATGAT

TCCTTCATATTTGCATATACGATACAAGGCTGTTAGAGAGATAATTGGAATTAATTTGACT

GTAAACACAAAGATATTAGTACAAAATACGTGACGTAGAAAGTAATAATTTCTTGGGTAG

TTTGCAGTTTTAAAATTATGTTTTAAAATGGACTATCATATGCTTACCGTAACTTGAAAGT

ATTTCGATTTCTTGGCTTTATATATCTTGTGGAAAGGACGAAACACCGGTAAATCGTGAT

gRNA 2 terminator

GCGACGTCAAGATTGTCGTTGTGGAAGGTCCAGTTTTGAGGGGCTATTACAACTTTTTTT

CTCCGCTGAGCGTACTGAGACGCCGCGGTGGAGCTCCAGCTTTTGTTC-3'

Figure S3 Partial DNA sequence of gRNA 2 expression plasmid (phU6-gRNA 2).

5'-TACCATGGTGATGCGGTTTTGGCAGTACATCAATGGGCGTGGATAGCGGTTTGACTC

ACGGGGATTTCCAAGTCTCCACCCCATTGACGTCAATGGGAGTTTGTTTTGGCACCAAA

CMV promoter

ATCAACGGGACTTTCCAAAATGTCGTAACAACTCCGCCCCATTGACGCAAATGGGCGGT

AGGCGTGTACGGTGGGAGGTCTATATAAGCAGAGCTCTCTGGCTAACTAGAGAACCCA

CTGCTTACTGGCTTATCGAAATTAATACGACTCACTACGGACAATCTTGACGTCGCATCA

CGATTTACCTCTCGCTCTCAGCACCATGGTGAGCAAGGGCGAGGAGCTGTTCACCGGG

GTGGTGCCCATCCTGGTCGAGCTGGACGGCGACGTAAACGGCCACAAGTTCAGCGTGT

CCGGCGAGGGCGAGGGCGATGCCACCTACGGCAAGCTGACCCTGAAGTTCATCTGCA

CCACCGGCAAGCTGCCCGTGCCCTGGCCCACCCTCGTGACCACCCTGACCTACGGCG

TGCAGTGCTTCAGCCGCTACCCCGACCACATGAAGCAGCACGACTTCTTCAAGTCCGC

EGFP

CATGCCCGAAGGCTACGTCCAGGAGCGCACCATCTTCTTCAAGGACGACGGCAACTAC

AAGACCCGCGCCGAGGTGAAGTTCGAGGGCGACACCCTGGTGAACCGCATCGAGCTG

AAGGGCATCGACTTCAAGGAGGACGGCAACATCCTGGGGCACAAGCTGGAGTACAACT

ACAACAGCCACAACGTCTATATCATGGCCGACAAGCAGAAGAACGGCATCAAGGTGAA

CTTCAAGATCCGCCACAACATCGAGGACGGCAGCGTGCAGCTCGCCGACCACTACCAG

CAGAACACCCCCATCGGCGACGGCCCCGTGCTGCTGCCCGACAACCACTACCTGAGC

ACCCAGTCCGCCCTGAGCAAAGACCCCAACGAGAAGCGCGATCACATGGTCCTGCTGG

AGTTCGTGACCGCCGCCGGGATCACTCTCGGCATGGACGAGCTGTACAAGAAGCTTAG

CCATGGCTTCCCGCCGGCTGTGGCTGCTCAGGATGATGGCACGCTGCCCATGTCTTGT

PEST

GCCCAGGAGAGCGGGATGGACCGTCACCCTGCAGCCTGTGCTTCTGCTAGGATCAATG

TGTAAGAATTCACTCGATATCTGCATCTAGAGGGCCCTATTCTATAGTGTCACCTAAATG

CTAGAGCTCGCTGATCAGCCTCGACTGTGCCTTCTAGTTGCCAGCCATCTGTTGTTTGC

BGH polyadenylation signal

CCCTCCCCCGTGCCTTCCTTGACCCTGGAAGGTGCCACTCCCACTGTCCTTTCCTAA-3'

Figure S4 Partial DNA sequence of target RNA 3 expression plasmid (pcDNA3-target 3).

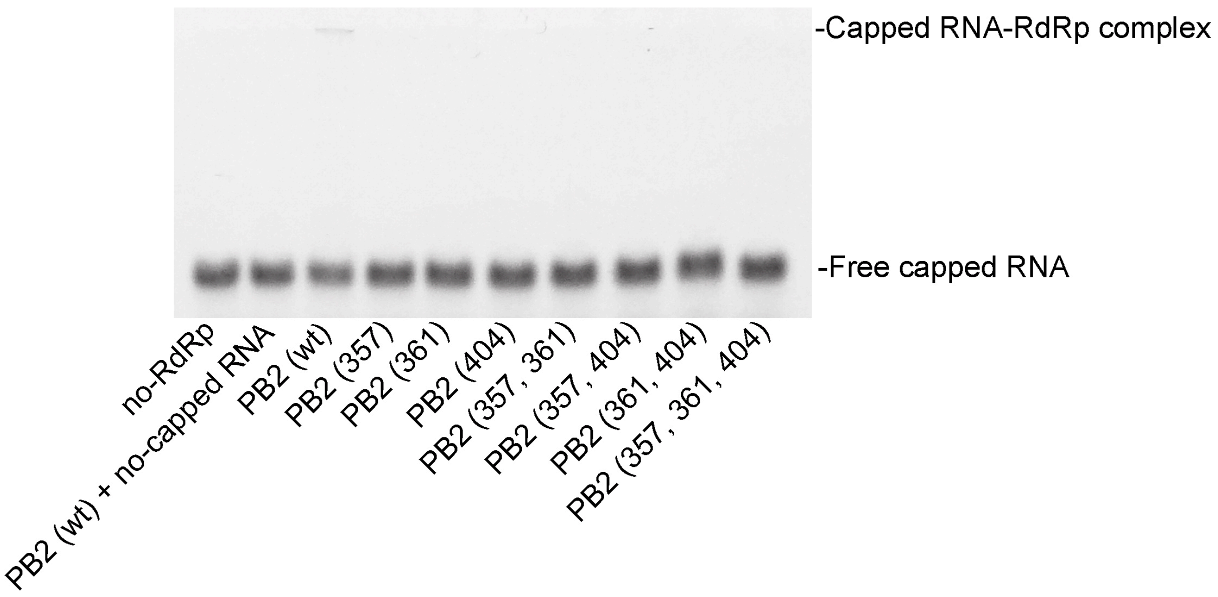

Figure S5 EMSA for interaction between 20-nt capped-RNA and RdRp containing PB2 mutant. EMSA was performed on a 4% native polyacrylamide gel in TBE buffer and run for 40 min with a field of 100 V at room temperature. Subsequently, gel was stained with GelGreen. Before loading, the mixture containing recombinant RdRp and 20-nt capped-RNA were incubated in reaction buffer (50 mM HEPES, pH 7.5, 150 mM NaCl, 1 mM DTT, 5 mM $MgCl_2$,) for 30 min at room temperature.

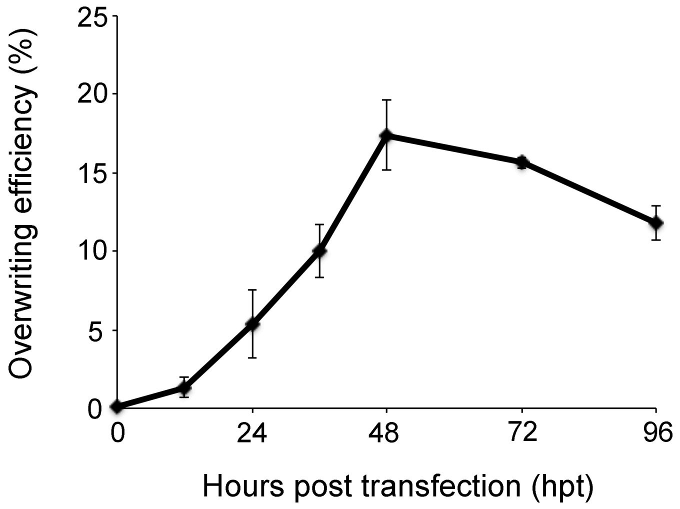

Figure S6 Time-course for RNA overwriting by PB2-dCas13b-containing RNP using gRNA 2 and target RNA 3. Overwriting efficiency was calculated in comparison to the case when RNA overwriting was performed using plasmid for expression of mScarlet-I mRNA instead of target EGFP mRNA.

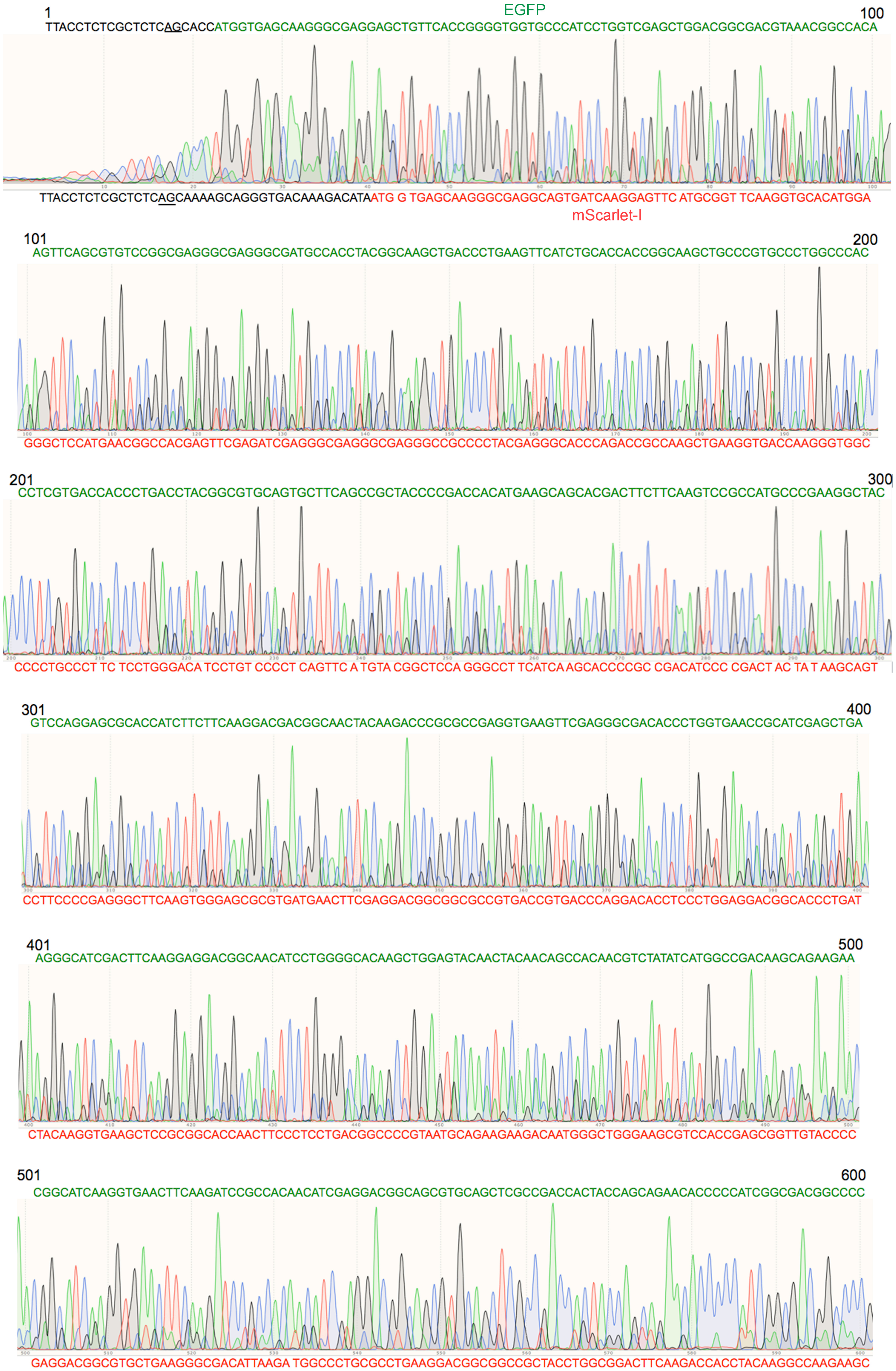

Figure S7 Sequence of overwriting products. The upper and lower sequences indicate the target and overwritten RNA, respectively. The underline represents the AG site, which is the initiation point of overwriting.

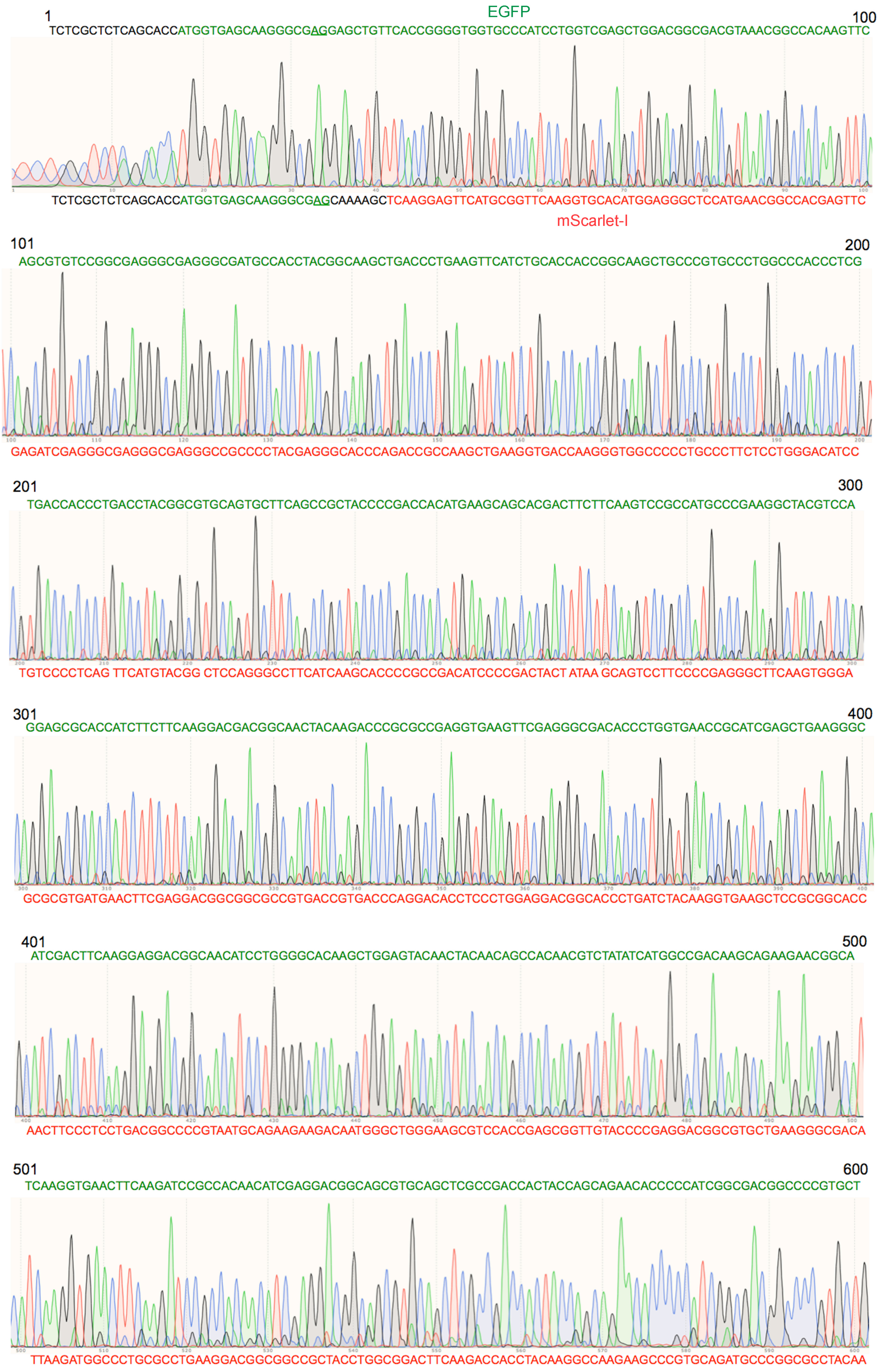

Figure S8 Sequencing data after overwriting in ORF. The upper and lower sequences indicate the target and overwritten RNA, respectively. The underline represents the AG site, which is the initiation point of overwriting.
